# Virulence is not directly related to strain success *in planta* in *Clavibacter nebraskensis*

**DOI:** 10.1128/msystems.01355-24

**Published:** 2024-11-29

**Authors:** Molly Veregge, Cory D. Hirsch, Matthew J. Moscou, Liana Burghardt, Peter Tiffin, Devanshi Khokhani

**Affiliations:** 1 Department of Plant Pathology, University of Minnesota, Twin Cities, Minnesota, USA; 2 USDA-ARS Cereal Disease Laboratory, University of Minnesota, St. Paul, Minnesota, USA; 3 Department of Plant Science, Pennsylvania State University, Center Valley, Pennsylvania, USA; 4 Department of Plant and Microbial Biology, University of Minnesota, Twin Cities, Minnesota, USA; E O Lawrence Berkeley National Laboratory, Berkeley, California, USA

**Keywords:** maize, select and resequence, *Clavibacter nebraskensis*, virulence, strain fitness

## Abstract

Goss’s wilt and leaf blight of maize is an economically important disease caused by the Gram-positive bacterium, *Clavibacter nebraskensis* (*Cn*). Little is known about the ecology and pathogenesis of this bacterium. Here, we used phenotypic assays and a high-throughput whole-genome sequencing approach to explore among-strain variation in virulence and multistrain reproductive success *in planta*. Our survey of 41 strains revealed that more recently sampled strains tended to have higher virulence than strains sampled before 2010 and tended to be more genetically divergent from the reference strain, isolated in 1971. More detailed assays with a representative sample of 13 of these strains revealed that host genotype (resistant or susceptible) did not strongly affect strain success and that strain success *in planta* in multi-strain communities was not closely associated with virulence in single-strain assays. Two weakly virulent strains, CIC354 and CIC370, had the greatest reproductive success, whereas the most highly virulent strains did not significantly change in frequency in any host genotype. A genomic analysis revealed candidate genes, including putative virulence factors (i.e., a secreted cellulase), responsible for among-strain variation in reproductive success.

**IMPORTANCE:**

Non-pathogenic strains of many bacterial pathogens are reported to coexist with pathogenic strains in symptomatic plants. To understand the ecology and pathogenesis of the pathogen population, it is essential to study strain dynamics in the context of the host. We created a community of 13 strains exhibiting diverse virulence phenotypes and used this community to infect the host plant. We compared the strain frequency of these strains before and after the host infection. Contrary to our hypothesis of highly virulent strains being selected by the susceptible host, we found that weakly virulent strains were selected by both resistant and susceptible host lines. We identified several genes associated with strain frequency shifts suggesting their role in strain colonization, virulence, and fitness.

## INTRODUCTION

Goss’s wilt and leaf blight (GWLB) is a severe bacterial disease of maize, causing up to 50% yield loss in susceptible varieties. This disease, caused by the Gram-positive bacterium *Clavibacter nebraskensis* (*Cn*), was highly destructive in the USA and Canada between 2016 and 2019 (1, 2). *Cn* infects maize tissue through openings caused by wounding, particularly from weather events such as hail or sandstorms, or through natural openings such as hydathodes (1, 3). When infected early, maize can exhibit systemic wilt symptoms (1, 4). Infection later in the season results in long, tan necrotic lesions on the leaf marked by characteristic areas of water-soaking known as “freckles” (1, 4). The pathogen overwinters in infected plant debris and can re-infect the crops the following year (1, 4). The adoption of low- or no-till farming practices paired with increased continuous corn cropping may have contributed to the re-emergence of GWLB as the pathogen can survive for 8–10 months in crop residue at the soil surface (1, 5). These agronomic practices have since made GWLB a significant threat to corn-growing regions in the United States and Canada.

*Cn* can colonize other prairie grass species and dicots like soybean without causing symptoms, hence affecting pathogen ecology (6). This phenomenon is not unique to *Cn*, as other pathogenic *Clavibacter* species have host ranges that include native species and crop plants (7). Uncharacterized and non-pathogenic *Clavibacter* species have been isolated from tomato, pepper, and maize, suggesting that multiple *Clavibacter* strains may coexist on one host (7, 8).

High-throughput sequencing technologies have been used to study microbial ecology and population dynamics within a multistrain community of *Sinorhizobia meliloti*, a facultative symbiont that colonizes *Medicago truncatula* and forms nitrogen-fixing nodules (9). This “select and resequence” approach operates by sequencing genomes of individual strains, forming a synthetic community of strains in approximately equal proportion, and using pooled sequencing and bioinformatic analyses to assess changes in relative strain frequency after exposure to selective pressure (i.e., host genotype). Burghardt et al. (2018) used select and resequencing to reveal that strain fitness within a multistrain community of *S. meliloti* was strongly influenced by host genotype and environmental selection (9, 10).

Our study uses the above approach to characterize interactions between *Cn* and maize. We expanded on previous studies that used single strains by incorporating *Cn* strains with a range of virulence phenotypes to investigate host-strain interactions (11–14). We investigated three major questions: (i) Do frequently occurring strains consistently outcompete other strains in every host genotype? (ii) Do resistant maize varieties show different patterns of strain frequencies post-infection from susceptible varieties? (iii) Does this have an effect on overall infection rates? Because avirulent strains can proliferate in the host in the presence of virulent strains as a result of nutrient leakage from host plant cells (15, 16), we also investigated the frequencies of weakly virulent and avirulent strains in the post-selected community to observe any positive selection of non-pathogenic strains.

Investigating these interactions lends insight into pathogen ecology and addresses key questions about which, if any, genetic traits undergo positive selection during infection. We hypothesized that (i) the most virulent *Cn* strains would be most strongly selected for during infection in susceptible hosts, (ii) post-selection communities would contain higher frequencies of allelic variants or genomic regions associated with virulence, and (iii) weakly virulent or avirulent strains would be positively selected by the host as a result of virulent strains providing increased nutrient reservoirs or achieving more effective host immune suppression or evasion.

## RESULTS

### Selection of representative strains of *Cn*

**Fig 1:**
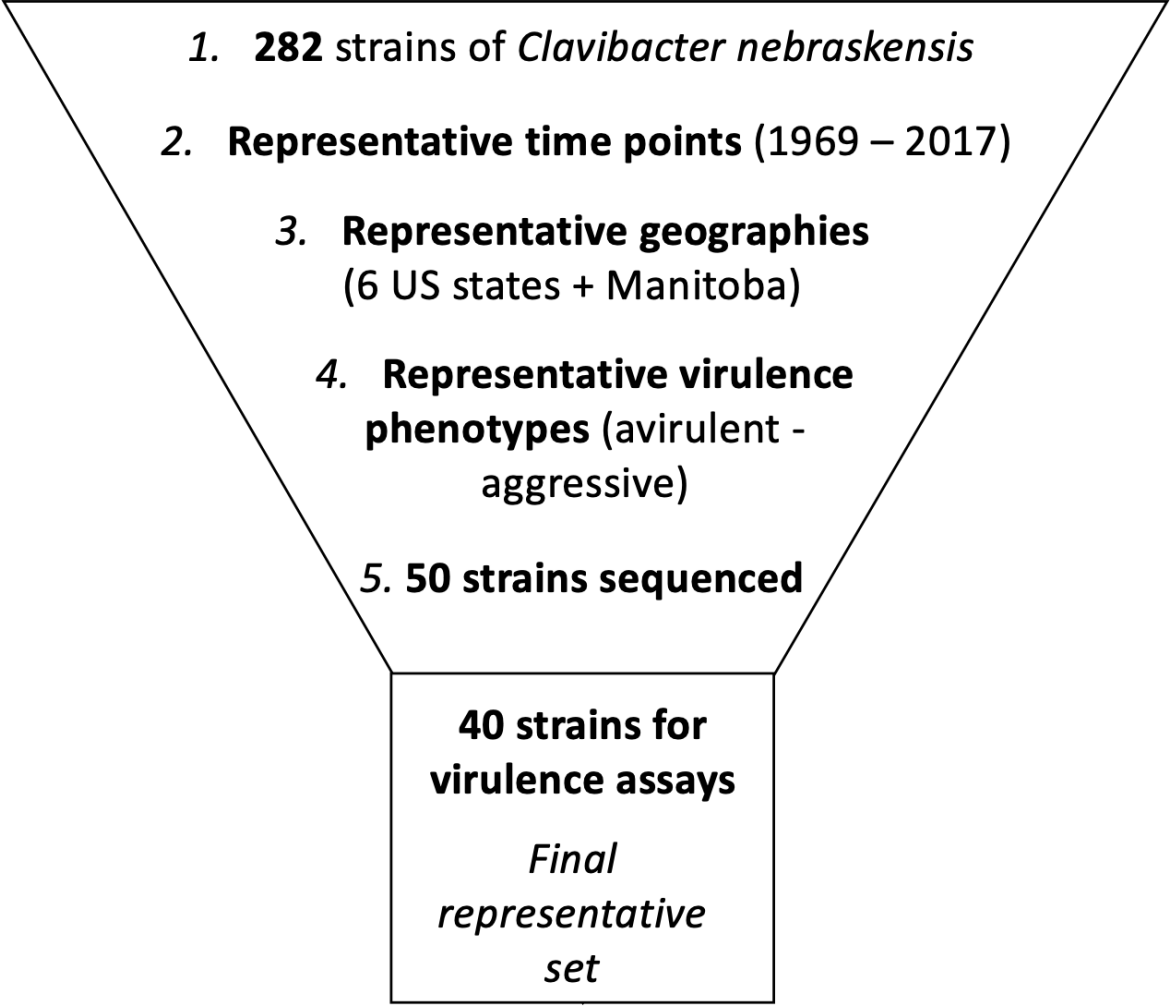
Selection process of 40 strains for virulence testing from a pool of 282 *Cn* strains. The date of collection, geographic location, and known virulence phenotypes were taken into account before sequencing. The final strains were chosen based on lack of sequence contamination and whole-genome SNP variability from the reference genome NCPPB 2581.

To identify a set of diverse *Cn* strains, we first evaluated the geographic origin, collection date, and known virulence phenotypes of the 282 *Cn* strains in our culture collection (Fig. 1). For strains that did not have any virulence phenotype data available, we chose a representative geography-date combination. Based on these data, we identified 50 strains with distinct geography-date-virulence profiles for Illumina whole-genome sequencing. From the resulting genomic data, we identified 40 strains that differed from the reference genome NCPPB 2581 (GenBank Accession HE614873) by at least 600 variable sites. We then assessed the virulence of these strains to confirm existing virulence phenotypes or to assign new virulence phenotypes (Fig. 1; Table 1).

**TABLE 1:** Virulence characteristics of 40 representative *Cn* strains^*a*^

Location	Number in collection	Number phenotyped	Dates across collection	Dates represented	Virulence
Colorado	8	4	1990–1998	1990	Weak–moderate
Iowa	30	4	2011–2013	2012–2013	Moderate–high
Minnesota	84	4	2009–2014	2009–2014	Moderate–high
Nebraska	59	10	1969–2012	1969–2012	Avirulent–high
North Dakota	26	11	2011–2017	2011–2017	Avirulent–high
South Dakota	7	2	2011–2017	2011	Moderate–high
Canada	68	5	2014–2017	2014–2017	Weak–high

^*a*^ The final *Cn* set was chosen to represent the diversity of dates of collection, geography, and known virulence types within the total collection.

### Virulence diversity of selected 41 *Cn* strains

**Fig 2:**
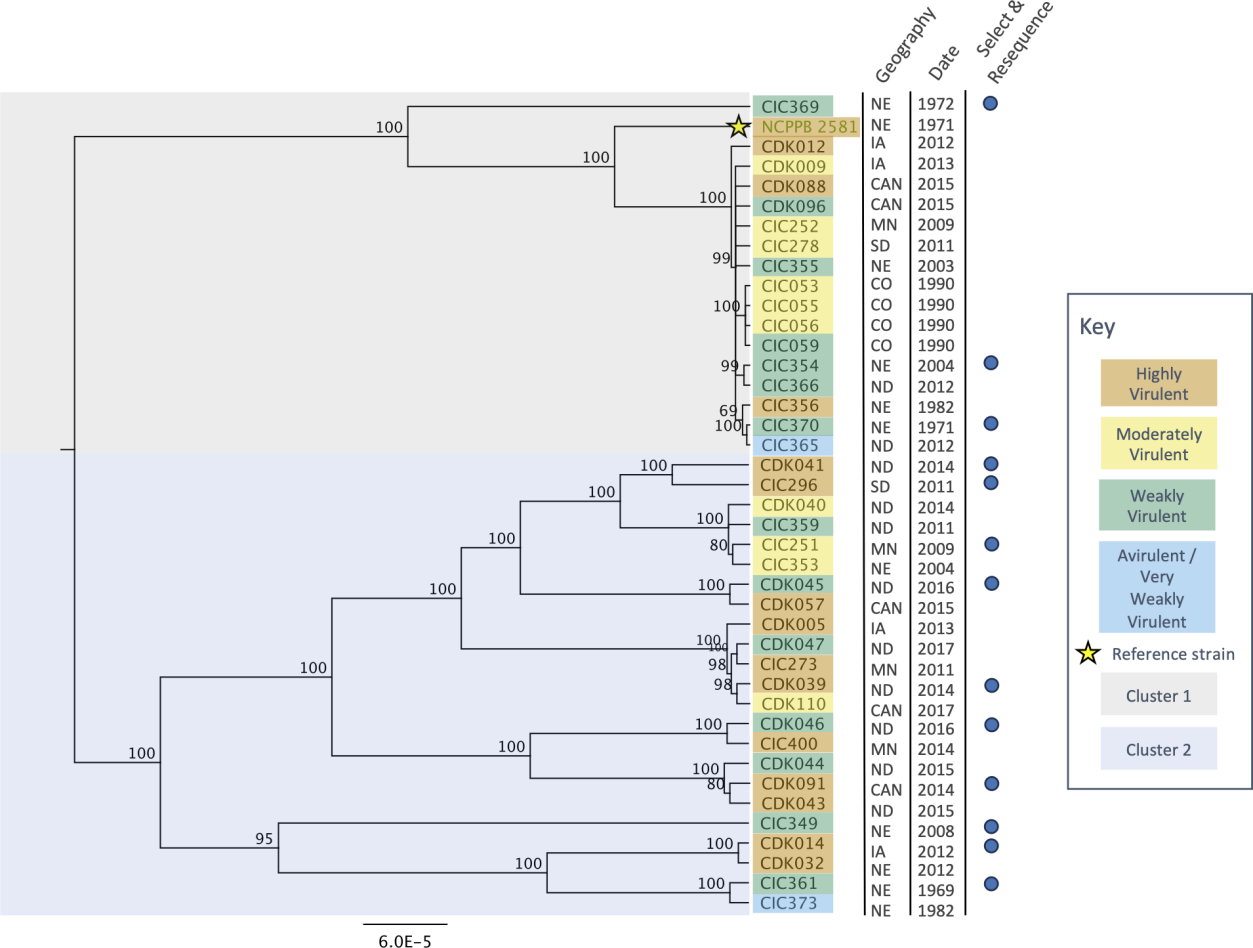
Phylogenetic tree of 40 *Cn* strains and reference strain, NCPPB 2581 (indicated with a yellow star), including geographic origin, date of collection, and the subset of strains chosen for select and resequence experiments. Boxes around phylogeny indicate clusters, and highlight colors indicate virulence score. Geography codes are as follows: CAN, Manitoba, Canada; CO, Colorado; IA, Iowa; MN, Minnesota; NE, Nebraska; ND, North Dakota; SD, South Dakota.

We assayed the above 40 *Cn* strains for virulence on the susceptible maize Inbred 34-1141 (17) and included the reference strain NCPPB 2581 (*syn*. CIC402) as a check for a total of 41 strains. Of the 41 strains phenotyped, 15, including the reference strain, were highly virulent (++, whole-tissue necrosis between 75% and 100%), 10 were moderately virulent (+, whole-tissue necrosis between 50% and 74%), 14 were weakly virulent (±, whole-tissue necrosis between 11% and 49%), and 2 were avirulent (−, whole-tissue necrosis between 0% and 10%) (Fig. 2; Table S1).

### Phylogeny of *Cn* strains

We examined the relatedness among the 41 *Cn* strains, including the reference strain, using whole-genome sequence data. We mapped Illumina sequencing reads from each strain onto the reference genome NCPPB 2581, identified single-nucleotide polymorphisms (SNP) segregating among the strains, and generated a phylogenetic tree. From this phylogeny, we identified two main clusters (Fig. 2), with Cluster 1 being considerably less diverse (601–1,221 variable sites) than Cluster 2 (2,122–3,765 variable sites). Cluster 1 contains 18 strains, including the reference strain, with 16 being very closely related within the same subcluster. Cluster 2 contains 23 comparatively diverged strains (Fig. 2). Relative to the strains in Cluster 2, the strains in Cluster 1, which includes the reference strain, tended to be older (mean sampling data 1997 vs 2010, median sampling date 2003 vs 2014, *Pt*-test <0.01), were less likely to be collected from more northerly locations (44% vs 78% collected from IA, ND, SD, MN, or CAN), and contained a higher percentage of strains ranked highly virulent (48% of Cluster 2 compared to 22% of Cluster 1). These data support the hypothesis that strains associated with later outbreaks in more northern regions are genetically distinct and more highly virulent than strains associated with earlier outbreaks (18).

### Select and resequence

#### Strain frequency shifts as a result of host selection

Of the 41 *Cn* strains shown in Fig. 2, we selected 13 strains that were representative of the range of phenotypic and genotypic diversity found. Our goal in selecting these 13 strains was to provide a broad and representative sample of the 41 phenotyped strains. Our final community of 13 strains contained fewer strains from Cluster 1 due to their high relatedness affecting downstream bioinformatic accuracy to differentiate these strains based on their SNP profiles. Similarly, bioinformatic analyses were unable to differentiate the avirulent strains CIC365 and CIC373 from related strains, so these were also eliminated from further analysis.

After identifying a diverse set of 13 isolates, we investigated strain dynamics in a multi-strain infection. To do this, we inoculated nine plants of each of four maize inbreds, two susceptible (Inbred 31-1141 and Oh7B), and two resistant (Mo17 and NC344) to GWLB with a pooled community of approximately equal frequencies of these 13 *Cn* strains (each comprising ~7% of the inoculum) and then estimated strain frequencies in each host at seven days post-infection.

**Fig 3:**
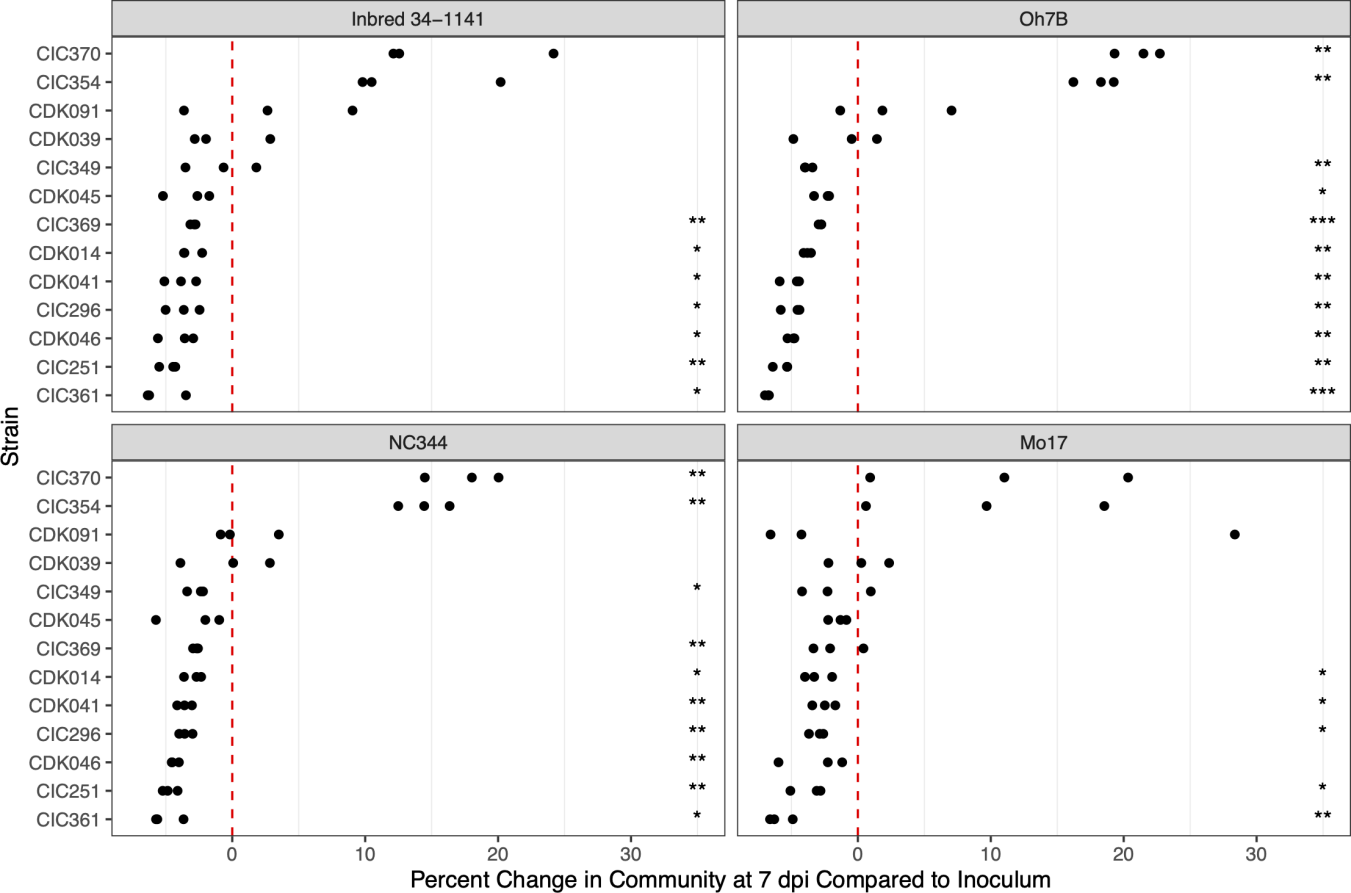
Percent change of *Cn* strains in the post-sequenced community at 7 days post-inoculation (dpi) compared with frequencies in the initial inoculum within each maize genotype. The plots of the maize genotypes are ordered top to bottom by susceptibility, with susceptible lines Inbred 34-1141 and Oh7B on top, and resistant lines NC344 and Mo17 on bottom. Significance was calculated by one-sample *t*-tests against a null percent change value of zero and is denoted by asterisks (*P < 0.05; **P < 0.01; ***P < 0.001).

We did not observe strong host genotype effects on strain frequencies. Across the four maize lines, the weakly virulent strains CIC354 and CIC370 became 2–4 times more prevalent after 7 days of growth within the host. CIC370 was the weakest virulent strain in the community, causing average tissue necrosis of 10%. The frequency of CIC370 increased from 8.0% to 9.1% in the initial inoculum to an average of 24.6% of the community at 7 dpi (an increase of ~16%). CIC354 caused an average tissue necrosis of 43% and comprised an average of 21.8% of the community at 7 dpi, compared to 7.8%–8.8% in the initial community (an increase of ~13.5%). In contrast, another weakly virulent strain, CIC361, which caused an average tissue necrosis of 21%, comprised only around 1.8% of the community at 7 dpi compared to 7.5% in the inoculum (a decrease of ~5.7%). The strains with the highest virulence scores, CDK091 that caused 94% average tissue necrosis and CDK039 that caused 92.5% average tissue necrosis, showed no significant differences in frequency in either direction (~3% increase and ~0.6% decrease in frequency, respectively). The remainder of strains showed reductions in prevalence in the community, with averages of 2.5%–5.8% reductions in frequency in the community at 7 dpi (Fig. 3).

**Fig 4:**
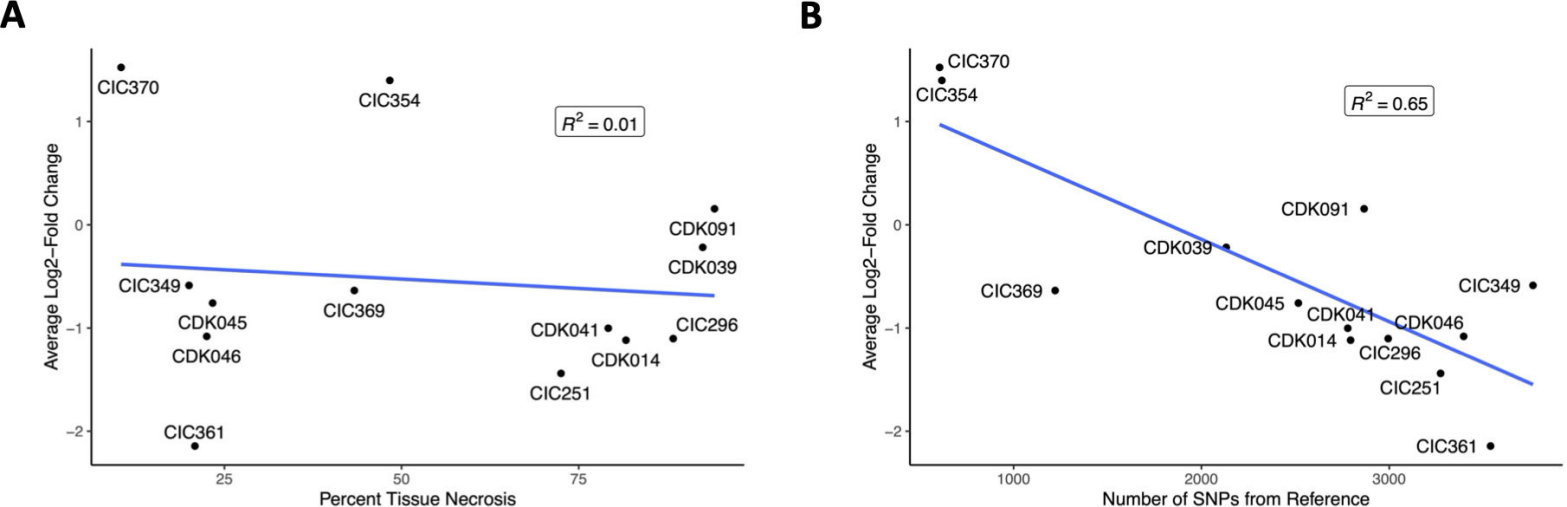
Correlation plots showing the relationship of virulence and genetic distance with selection within hosts. The correlation plot of virulence (denoted by percent tissue necrosis) against selection (denoted by average log2-fold change across all hosts) shows no correlation between virulence and selection (**A**). The correlation plot of genetic distance (denoted by the number of SNPs from the reference genome) against selection (denoted by average log2-fold change) shows a moderately high correlation between genetic distance from the reference and selection (**B**).

We performed correlation analyses to identify variables strongly associated with selection in the plant host. We found no strong relationship between virulence and strain frequency at 7 dpi, indicating that virulence did not play a role in community selection (*R*^2^ = 0.01, Fig. 4A). By contrast, we detected a moderately strong correlation between the number of variable sites from the reference genome and selection in the plant host (*R*^2^ = 0.65, Fig. 4B).

#### Genomic regions associated with strain frequency shifts

**Fig 5:**
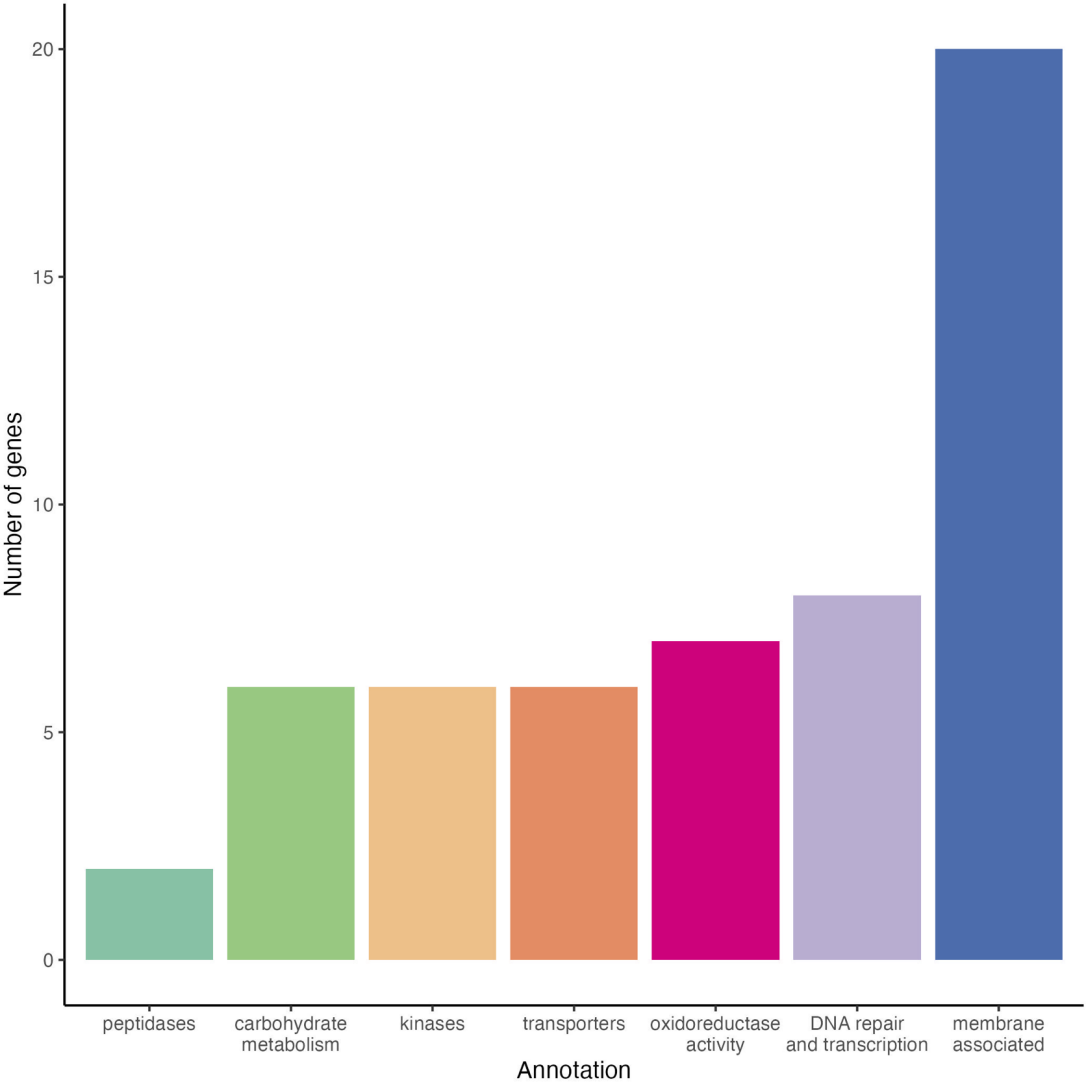
Summary of annotations of top gene candidates involved in *Cn* host selection identified through Fisher’s exact test. Gene ontology (GO) annotations from candidate genes were derived from UniProt entries and binned into classes based on the most frequently occurring terms in GO annotations, including “peptidase,” “carbohydrate metabolism,” “kinase,” “transporter,” “oxidoreductase,” “DNA repair,” “transcription,” and “membrane.”

We performed a Fisher’s exact test to identify SNPs under selection *in planta* (i.e., associated with changes in strain frequency). With a limited sample size and to reduce the chances of false positives, we considered SNPs in the top 0.5% of −log10 *P*-values identified by Fisher’s exact test as candidates. This analysis identified 202 SNPs associated with 94 genes, 77 of which had annotated gene products (Table S3) and 71 of which resulted in missense variants within 51 genes (Table S4). While positive selection *in planta* did not correlate with strain virulence, a subset of the genes identified by the Fisher’s exact test have a putative function that is likely to contribute to pathogenicity (Fig. 5).

Genes under selection showed a range of functions. We identified 55 of the 77 annotated genes that had GO terms including “carbohydrate metabolism,” “DNA repair” or “transcription,” “kinase,” “membrane,” “oxidoreductase,” “peptidase,” and “transporter.” Although membrane-associated genes appeared in a high frequency in this set, enrichment analyses did not reveal an overrepresentation of membrane-associated genes. However, GO term enrichment analyses revealed an overrepresentation of genes with biological functions associated with DNA damage repair, lipid metabolic processes, and terpenoid biosynthesis, and molecular functions associated with catalytic and transferase activity (Fig. S1).

Genes with GO term functions including carbohydrate metabolism included two pectinesterases, *bglY* encoding a glycosyl hydrolase, *xysB* encoding Endo-1,4-beta-xylanase, *manX* encoding a putative alpha-mannosidase, and *cel* encoding a secreted cellulase (Fig. 5; Table S2). Due to the known importance of CAZymes in phytobacterial pathogenicity, we analyzed sequence types constructed from unique SNP combinations found in our population to explore relationships between sequence types and virulence in these putative virulence factors. We found eight alleles within the merged pectinesterase genes, which appeared in one locus on the genome at CMN_00249 and CMN_00250. However, alleles did not correlate with virulence, and strains that were highly virulent shared sequence types with weakly virulent strains. Three alleles were observed for *bglY*, four within *xysB*, and six within *cel*, none of which correlated with virulence phenotype. Concatenating these four CAZyme loci under selection led to 12 distinct sequence types. The only sequence type shared between strains was between a highly virulent and weakly virulent strain, leading to no elucidation of sequence types associated with virulence. However, closer inspection of CMN_00249, the first pectinesterase gene in the pectinesterase locus, trends toward a lack of polymorphisms associated with virulence. Sixty percent of highly virulent strains contained no SNPs from the reference genome in this gene, whereas only 29% of weakly virulent strains contained no SNPs. Regardless, the presence of 12 unique sequence types within 13 strains at these 4 loci highlights the considerable genetic diversity present within this synthetic pathogen community.

## DISCUSSION

We performed a select and resequence experiment using a consortium of 13 *Cn* strains with varying virulence isolated from diverse geographical locations and across approximately four decades. We found that two weakly virulent strains (CIC354 and CIC370) increased in frequency, becoming 2–4 times more prevalent in the community at 7 dpi (211%–402% of their initial frequencies). Surprisingly, the most highly virulent strains in the community did not show any significant changes in frequency at 7 dpi, while the remaining strains were present at just 24%–70% of their initial frequencies in the community at 7 dpi.

### Strain selection across diverse maize genotypes

Although the reproductive success of strains varied *in planta*, strain success did not differ among host genotypes. Host resistance to Goss’s Wilt is mainly controlled by many small-effect QTL (11, 12). Unlike qualitative resistance, which is highly race-specific and controlled by one or a few genes, quantitative resistance is broad, enforcing less selective pressure on the pathogen population.

Contrary to our hypothesis, virulent strains were not the most highly selected (Fig. 4A), and the strain with the lowest virulence score in the pool (CIC370) was consistently positively selected. Several mechanisms, other than virulence, might be responsible for among-strain differences in reproductive success *in planta*. One possibility is that some strains act as “cheaters,” procuring nutrients leaked from degraded plant cells as a result of virulence factors secreted by other strains. This could allow some weakly virulent strains to proliferate more quickly in the plant host than highly virulent strains, as they are able to uptake essential nutrients without the fitness cost of producing secreted virulence proteins, such as toxins or cell wall-degrading enzymes. However, while two weakly virulent strains became more frequent at 7 dpi, one weakly virulent strain decreased in frequency to the highest magnitude compared to all other strains. Additionally, the ability of strains to evade the host immune response could play a role in their success *in planta*. Maize has been shown to produce ROS as a response to infection by *Cn,* and the ability of a strain to escape oxidative pressures by neutralizing these ROS could play a role in their success (13). For example, Soliman et al. (2021) found that many proteins with antioxidant activity were significantly upregulated in the secretome of *Cn* grown in xylem sap (19).

Alternatively, bacteriocin production in *Cn* could play a role in competition. However, bacteriocins within the genus *Clavibacter* are generally effective against other species in the genus but are only marginally effective on other strains within the species (20). Additionally, bacteriocin production in *Cn* has been shown to be relatively homogeneous, with the production of two main bacteriocins, CN1 and CN2 (21). These bacteriocins show efficacy against other *Clavibacter* species, but *Cn* strains tested against one another showed no sensitivity to CN1 and variability in sensitivity to CN2 (21). It is possible that strains from more recent epidemics could show new bacteriocin diversity, and under this hypothesis, strains with higher reproductive success in the community at 7 dpi could secrete newly active bacteriocins against other competing strains. However, this hypothesis would require further experimentation via *in silico* prediction of bacteriocins complemented by pairwise testing of all strains *in vitro* to assess competition at a strain-by-strain level. Therefore, the exact relationship between weakly and highly virulent strains remains unclear. Investigating the secretome of both highly and weakly virulent strains would provide clarity on which strains are most dominantly influencing the apoplastic environment through the production of virulence factors.

Interestingly, the trait most strongly associated with strain selection was the genetic distance from the reference strain (*R*^2^ = 0.65, Fig. 4B). We generated two hypotheses for this relationship, one technical and one biological. The technical possibility we considered was that a bias in the software used in bioinformatic analyses disproportionately identified strains that were more similar to the reference strain. However, analyses of simulated read pools revealed high correlations between known frequencies of strains in simulated read pools and the estimated frequencies generated by HARP (22), thereby rejecting this possibility (Fig. S2). The biological possibility is that the type strain NCPPB 2581, isolated in 1971 and used as the reference strain in this experiment, is the most adapted to its host and that genomic differences from this strain led to lower competitive advantages in a multistrain community. In our study, the number of variants from the reference across the genome does not correlate with virulence. Similarly, in past analyses, SNP variations in putative virulence genes also do not tend to correlate with virulence phenotype (18, 23). This points to a complex underpinning of virulence in these diverse strains that cannot be explained by a simple linear model of virulence based on SNP profiles. Our data did not validate the hypothesis that highly virulent strains would be most highly selected for during infection, leading to new questions pertaining to what shapes strain fitness in the host and how strains interact and compete within a host plant. There may also be antagonistic microbes in the environment that play a role, of which we have little knowledge. Additionally, we hypothesized that if highly virulent strains were positively selected, a resulting shift in allele frequencies of putative virulence factors would lend insight into what genomic elements are associated with virulence. However, the positive selection of weakly virulent strains leads us to disentangle the questions of virulence and fitness within a host.

### Genomic regions under selection in *Cn*

Six genes associated with carbohydrate metabolism were under significant selection in the maize host, including two pectinesterases, *bglY* encoding a glycosyl hydrolase, *xysB* encoding Endo-1,4-beta-xylanase, *manX* encoding a putative alpha-mannosidase, and *cel* encoding a secreted cellulase (Fig. 5; Table S2). While some genes could simply be related to strain growth across diverse substrates, in closely related *Clavibacter* species, secreted cellulases are required for virulence (24–26). However, SNPs within these putative virulence genes in *Cn* did not correlate with virulence, and their functional role in supporting strain growth is not known (18, 23). Similarly, we found no relationship between sequence types constructed from SNP profiles and levels of virulence in our community.

DNA repair mechanisms and oxidative stress responses are important to bacterial cells under stress from plant defense responses, including the production of reactive oxygen species (ROS). Therefore, the selection of bacterial genes involved in DNA repair and stress response could play a role in protecting *Cn* strains from the defense responses of the host plant. Maize defense to GWLB has been linked to the salicylic acid (SA) and programmed cell death (PCD) responses, which require the production of reactive oxygen species to trigger defense signaling (13). Selection of bacterial genes such as the molecular chaperone *dnaK*, the DNA repair genes *xthA* and *ogtB*, and the DNA polymerase *polA2* under infection conditions could indicate bacterial stress response, including DNA repair and protein refolding, in response to maize ROS production. *dnaK* has been implicated in stress response in both plant and animal bacterial pathogens and contributes to the full virulence of phytopathogenic *Xanothomonas* species (27, 28). Additionally, Soliman et al. (2021) noted the upregulation of *dnaK* in the secretome of *Cn* infecting maize, supporting the putative role of this molecular chaperone in protein refolding as a response to oxidative stress in *Cn* (19).

Transcriptional rewiring is also a key bacterial response to stress. The identification of four genes involved in transcriptional regulation could signal involvement in transcriptional rewiring associated with stress, pathogenesis, or downstream carbohydrate utilization (Table S2). Twelve genes with kinase domains or ABC-transporter annotations were also identified, indicating selection associated with signal transduction and transport (Fig. 5; Table S2).

Interestingly, a large portion of SNPs associated with changes in strain frequency (22%) were in genes annotated with the GO term “membrane,” including *wcoC* involved in lipopolysaccharide synthesis (Table S2). LPS production is associated with biofilm formation and virulence in bacterial phytopathogens. Bacterial pathogens must successfully adhere to plant cells and establish sufficient populations in order to initiate infection, which is enabled by the production of exopolysaccharides. Additionally, biofilm formation could play a role in establishing a protective layer around bacterial cells exposed to ROS or defense-related proteins produced by the host cell, which could explain its role in the proliferation of less virulent strains *in planta*. In the context of establishing infection, *Cn* invades the host maize plant through hydathodes, subsequently colonizing the xylem and then moving into the mesophyll (3). Cell attachment and subsequent biofilm formation are essential to establish sufficient cell numbers in the host to initiate degradation of host cell walls by bacterial phytopathogens, and in the case of *Cn*, biofilm formation in the xylem is likely required before movement into the mesophyll. Therefore, the putative involvement of *wcoC* represents an imperative step in the colonization of xylem cells by *Cn*. Of note, *wcoC* belongs to the same protein family as *gumD* in *Xanthomonas*, a gene shown to be important for biofilm formation and virulence (29, 30).

Peptidases are also a key player in bacterial-mediated pathogenesis. Secreted bacterial proteases can cleave peptide bonds in the plant cell wall, facilitating colonization, and subsequent infection. They can also mediate interactions between the bacterium and plant defense systems, as well as remove any misfolded or denatured proteins as a result of attack by the plant immune response (31). Two peptidases were identified as alleles under selection: CMN_00051, a pyroglutamyl peptidase (cysteine protease), and *pepP2*, an Xaa-Pro aminopeptidase. Cysteine proteases have been shown to act as effectors in Gram-negative bacteria (32). However, in *Clavibacter* spp., the role of serine proteases has been more closely linked to function in pathogenicity and virulence (33–36). The role of this particular pyroglutamyl peptidase in pathogenicity is currently unknown and will require further investigation. Of note, *pepP2* was also identified as a differentially expressed protein in the secretome of *Cn* by Soliman et al. (2021), supporting the role of this protein in the pathogenesis of *Cn* (19).

### Conclusion

This study employed high-throughput whole-genome sequencing to explore *Cn* diversity and ecology with more granularity. Using a select and resequence approach, we found that host genotype did not significantly influence the selection of *Cn* strains likely due to the underlying quantitative resistance in maize (11, 12). Surprisingly, the most selected strains, CIC354 and CIC370, were weakly virulent in single-strain inoculations, suggesting that complex community dynamics influenced selection more than virulence. Our findings indicate that weakly virulent strains might act as “cheaters” by utilizing resources produced by highly virulent strains or that bacteriocin production might influence competition among strains, necessitating further functional studies to validate this hypothesis. Variants in genes related to virulence factors such as CAZymes, DNA transcription and repair, and oxidative stress response were associated with changes in strain frequencies. Further experimentation is needed to understand the role of these alleles in strain colonization, fitness, and virulence.

## MATERIALS AND METHODS

### Strain selection and single-strain whole-genome sequencing

The original subspecies of *C. michiganensis sensu lato* are elevated to the species level, thus the Goss’s wilt pathogen, previously known as *Clavibacter michiganensis* subsp. *nebraskensis* was designated as *Clavibacter nebraskensis* (37). Strains from a culture collection of 282 *C*. *nebraskensis* strains isolated from 7 US states and Mantiboa, Canada, between 1969 and 2017 were reviewed and 50 representative strains were chosen for whole-genome sequencing based on geographic origin, date of collection, and virulence phenotype. Cultures were grown on NBY agar with or without cycloheximide for 5–7 days at 25°C, and then genomic DNA was extracted with the DNeasy UltraClean Microbial Kit (Qiagen) according to the manufacturer’s protocol. The purity and concentration of gDNA were checked with a nanodrop, and then samples were submitted to the University of Minnesota Genomics Center (UMGC). Libraries were prepared with the Illumina DNA Flex Kit and paired-end sequenced on an Illumina MiSeq with v3 chemistry (300 cycles). The data files associated with whole-genome sequencing are submitted to Sequence Read Archive on NCBI (Bioproject ID—PRJNA1131012) which will be released upon publication.

### Phylogenetic analyses of *C. nebraskensis*

Sequencing data obtained from the UMGC were demultiplexed, and quality was checked using FastQC (v0.11.7). Adapter contamination and poor-quality reads (quality <30) were trimmed using Trimmomatic (v0.33), with the lower threshold of read length set to 150 base pairs. Reads were then aligned to the type strain reference genome *Clavibacter michiganensis* subsp*. nebraskensis* strain NCPPB 2581 (GenBank Accession HE614873) using BWA (v0.7.15) and SAMtools (v1.9). Variant calling was then performed with BCFtools (v1.9). We then removed any contaminated samples, identified by low sequencing coverage and percentage of mapped reads, and strains that differed from the reference genome at <600 nucleotide sites. After removing these samples, we were left with 40 strains for subsequent analyses.

Phylogenetic analyses were performed on the resulting merged vcf file of these 40 strains using Geneious Prime (v2022.2). An Unweighted Pair Group Method with Arithmetic Mean (UPGMA) consensus tree using a Jukes-Cantor genetic distance model was generated, with 100 bootstrap replicates and a support threshold of 50%.

### Single-strain virulence assays

We tested the virulence of the 40 strains used for genomic analyses on the susceptible maize line Inbred 34-1141 (17). Two maize seeds were sown in D60L lightweight Deepot cell containers (983 mL) filled with SunGro potting soil amended with Osmocote 15-9-12, Sprint 330 chelated iron, and ferrous sulfate. After germination, we thinned seedlings to one seedling per pot. Seedlings were watered every two days or as needed and fertilized once a week with a 15-5-15 Cal Mag special. Seedlings were inoculated at growth stage V3. Inoculations were carried out in triplicate in a Conviron growth chamber on a 16 h light (25°C) and 8 h dark (23°C) cycle with 25% humidity and repeated once for a total of two trials. Inoculum was created by suspending *Cn* cells grown on NBY agar for 5–7 days at 25°C in 1× phosphate-buffered saline (PBS) and adjusted to 10^8^ CFU/mL. A stab inoculation method was used, where a 1 mL syringe with a needle was inserted into the stem approximately 2.5 cm above the soil line and about 1 cm deep, and 100 µL of inoculum was injected into the wound. Then, a similar wound perpendicular to the initial wound was created and another 100 µL of inoculum was injected for a total of 200 µL of inoculum per plant. Plants were scored for symptoms at 3, 5, and 7 days post-inoculation (dpi), and scores based on the percentage of whole plant tissue necrosis were assigned according to the previously designed scheme (18).

### Select and resequence experiment

#### Community strain selection

A subset of 13 *Cn* strains was chosen as the final community for select and resequence analyses. We selected 13 strains that represented early and late collection times (1969–2016), geographic origins (5 US states and Manitoba, Canada), and virulence levels (weakly virulent to highly virulent) (Fig. 2). An avirulent strain was not chosen as part of the community because the software used for bioinformatic differentiation of strains, HARP [Haplotype Analysis of Reads in Pools (22)], was unable to detect an avirulent strain in pooled reads. The accuracy of HARP in predicting strain frequencies was verified by creating simulated mock communities by sampling sequencing reads from individual strains in known frequencies and merging them into a single FASTA file, then running HARP and correlating output frequency files with known frequencies. The final selection of the 13 strains used for this experiment was based on validation experiments of the HARP software to accurately identify reads originating from individual strains using simulated read pools from whole-genome sequencing data.

#### Inoculation

Four maize inbred lines, two susceptible (34-1141 and Oh7B) and two resistant (Mo17 and NC344), were chosen for analysis (3, 17). Seedlings were grown as described above and inoculated at the V2 growth stage with 13 strains in approximately equal proportions. Strains were prepared by growing each on NBY for 5 days at 27°C, then suspending in 1× PBS and adjusting cell density to 10^8^ CFU/mL, and then equal volumes of each strain were mixed together to ensure equal proportions of each strain in the final community to offset any differences in *in vitro* growth rates. A subset of this inoculum was saved for DNA extraction by centrifuging at 3,000 × *g*, removal of the supernatant, and freezing at −20°C.

#### Tissue collection, DNA extraction, and sequencing

Infected maize leaf tissues were collected at 7 dpi and flash-frozen in liquid nitrogen. Samples were stored at −80°C until extraction. Tissue samples were ground via mortar and pestle in liquid nitrogen, then DNA was isolated using a CTAB and phenol/chloroform extraction method (38). Three samples of each genotype were pooled to comprise one biological replicate. Samples were then submitted to UMGC for library preparation and sequencing. For samples that did not meet quality control criteria, sample cleanup was performed with the New England BioLabs Monarch PCR and DNA Cleanup Kit (5 µg), and clean samples were resubmitted to UMGC.

DNA from pooled inoculum that had been stored at −20°C was extracted with the same CTAB and phenol/chloroform extraction method (38). The purity and concentration of gDNA were checked with a nanodrop, and then samples were submitted to UMGC for library preparation and sequencing. Libraries were prepared with the Illumina DNA Flex Kit, and paired-end sequenced on an Illumina NovaSeq SPrime (150 cycles). Sequencing reads were checked for quality using FastQC (v0.11.7). Adapters and low-quality sequences were trimmed using Trimmomatic (v0.33) with a minimum quality parameter set to 30, a minimum sequence length threshold of 100 bp, and a minimum overlap with adapter sequences set to 3.

Trimmed reads were aligned to the reference genome *Clavibacter michiganensis* subsp*. nebraskensis* strain NCPPB 2581 (GenBank Accession HE614873) using bwa mem (v0.7.15). Samtools (v1.9) was used to generate sorted bam files. From these bam files, we generated a merged vcf file, filtered for only SNP sites, and identified 5,830 SNPs which we used to estimate strain frequency in pooled reads using HARP (22).

#### Statistical analysis

The percent change of strain frequency in post-sequenced communities was calculated by taking the difference of the strain percentage in communities at 7 dpi by the strain percentage in the initial inoculum, predicted by HARP. The probability of this percent change occurring by chance was estimated by performing *t*-tests on percent difference values of each strain against a null value of zero.

#### Measurement of allele frequencies

From bam files of initial pools and post-infection pools from each genotype, merged bam files of initial pools and post-infection pools were created using samtools (v1.9). A pileup file was generated for these bam files and converted to a sync file for use with PoPoolation2. Fisher’s exact test was calculated to measure the significance of allele frequency changes in all post-infection pools compared to both initial pools, and the resulting data were plotted on a Manhattan plot using ggplot2 in R (v4.3.1) (Fig. S3). Due to a small sample size to reduce false positives, we considered variants with a −log10 *P*-value in the top 0.5% as being candidates for contributing to changes in strain frequencies. To determine SNP effects within coding regions, we used SnpEff (v5.1d) to analyze our filtered vcf file.

## SUPPLEMENTAL MATERIAL

Supplemental material msystems.01355-24-s0001.pdf Supplemental tables and figures.

ASM does not own the copyrights to Supplemental Material that may be linked to, or accessed through, an article. The authors have granted ASM a non-exclusive, world-wide license to publish the Supplemental Material files. Please contact the corresponding author directly for reuse.
