# Supplementary material for: Virulence is not directly related to strain success in planta in Clavibacter nebraskensis
Source: mSystems. 2024 Nov 29;10(1):e01355-24. doi: 10.1128/msystems.01355-24 (PMC11748494; doi:10.1128/msystems.01355-24)
Supplement: Supplemental material — Supplemental tables and figures. [file msystems.01355-24-s0001.pdf]

Supplementary Information

Table S1. Virulence scores, phylogenetic cluster membership, geography, and date of collection of the forty-one phenotyped *Cn* strains, including the reference strain NCPPB 3581 (listed as CIC402).

Strain	Score	Cluster	Location	Date
CDK005	++	2	IA	2013
CDK009	+	1	IA	2013
CDK012	++	1	IA	2012
CDK014	++	2	IA	2012
CDK032	++	2	NE	2012
CDK039	++	2	ND	2014
CDK040	+	2	ND	2014
CDK041	++	2	ND	2014
CDK043	++	2	ND	2015
CDK044	+/-	2	ND	2015
CDK045	+/-	2	ND	2016
CDK046	+/-	2	ND	2016
CDK047	+/-	2	ND	2017
CDK057	++	2	CAN	2015
CDK088	++	1	CAN	2015
CDK091	++	2	CAN	2014
CDK096	+/-	1	CAN	2015
CDK110	+	2	CAN	2017
CIC053	+	1	CO	1990
CIC055	+	1	CO	1990
CIC056	+	1	CO	1990
CIC059	+/-	1	CO	1990
CIC251	+	2	MN	2009
CIC252	+	1	MN	2009
CIC273	++	2	MN	2011
CIC278	+	1	SD	2011
CIC296	++	2	SD	2011
CIC349	+/-	2	NE	2008
CIC353	+	2	NE	2004
CIC354	+/-	1	NE	2004
CIC355	+/-	1	NE	2003
CIC356	++	1	NE	1982
CIC359	+/-	2	ND	2011
CIC361	+/-	2	NE	1969
CIC365	-	1	ND	2012

CIC366	+/-	1	ND	2012
CIC369	+/-	1	NE	1972
CIC370	+/-	1	NE	1971
CIC373	-	2	NE	1982
CIC400	++	2	MN	2014
CIC402	++	1	NE	1972

Table S2. Quality control of the fifty sequenced *Cn* strains. Strains with significant contamination, identified here as strains with low sequencing depth and low percentage of reads that mapped to the reference genome, were removed from further analyses. Additionally, strains with less than 600 variable sites from the reference genome or that were deemed duplicate strains were removed from further analyses.

Strain	Sequencing depth	Percent of reads that mapped to the reference	Number of variants	Excluded from further analysis
CDK003	12.1	44.6	8194	X
CDK005	26.2	99.3	2150	
CDK008	2.2	18.0	37668	X
CDK009	26.9	99.9	636	
CDK012	26.1	99.9	624	
CDK014	26.9	98.8	2794	
CDK015	18.7	85.3	170034	X
CDK028	2.1	17.6	38318	X
CDK029	2.4	18.7	38885	X
CDK032	24.1	95.2	2832	
CDK034	2.1	18.1	37590	X
CDK039	25.5	93.0	2132	
CDK040	28.7	99.1	3333	
CDK041	24.9	99.2	2779	
CDK043	27.4	98.8	2836	
CDK044	24.9	97.0	2828	
CDK045	26.5	96.9	2516	
CDK046	27.0	98.7	3396	
CDK047	24.5	96.8	2164	
CDK057	22.8	99.2	2461	
CDK060	25.3	100.0	26	X
CDK088	23.9	99.9	615	
CDK091	25.0	98.9	2866	
CDK096	28.1	99.9	622	
CDK110	27.0	99.3	2149	

CIC012	23.0	86.8	73	X
CIC053	30.9	99.9	616	
CIC055	23.1	99.9	616	
CIC056	28.5	99.8	613	
CIC059	29.8	99.9	601	
CIC251	26.6	99.3	3273	
CIC252	27.3	99.9	619	
CIC273	25.4	96.2	2122	
CIC276	0.2	4.0	1467	X
CIC278	30.2	99.9	615	
CIC296	24.4	95.7	2994	
CIC346	28.4	99.9	607	X
CIC349	27.3	98.3	3765	
CIC353	29.1	99.1	3302	
CIC354	25.1	99.2	618	
CIC355	27.0	99.9	621	
CIC356	29.5	99.9	602	
CIC359	24.2	99.1	3285	
CIC361	25.8	98.0	3539	
CIC365	25.7	99.8	607	
CIC366	23.1	99.2	618	
CIC369	19.8	99.9	1221	
CIC370	23.8	99.9	606	
CIC373	24.1	95.4	3521	
CIC400	26.4	98.7	3361	

Table S3. Genes containing alleles under the highest selective pressure in the *Cn* genome during infection of maize hosts. A diversity of gene functions is represented, with genes of note including carbohydrate-active enzymes, genes associated with transcription and DNA repair, oxidative stress response genes, membrane-associated proteins, and transporters. Locus tags are from strain NCPPB 2581 (GenBank Accession HE614873).

Locus	Gene Symbol	Annotated Gene Product	GO Terms	GO Term Annotations
CMN_00039		conserved hypothetical protein		
CMN_00047		short-chain dehydrogenase/reductase	GO:0016491	oxidoreductase activity
CMN_00050		conserved hypothetical protein		
CMN_00051		pyroglutamyl-peptidase I		
CMN_00065	*dnaK*	Chaperone protein dnaK	GO:0005524<br>GO:0140662<br>GO:0051082	ATP binding<br>ATP-dependent protein folding chaperone<br>Unfolded protein binding
CMN_00083		hypothetical protein		
CMN_00089		conserved membrane protein	GO:0016020	membrane
CMN_00093		two-component system, histidine kinase	GO:0016020<br>GO:0000155<br>GO:0046983	membrane<br>phosphorelay sensor kinase activity<br>protein dimerization activity
CMN_00174		hypothetical membrane protein	GO:0016020	membrane
CMN_00249		pectinesterase	GO:0045330<br>GO:0016798<br>GO:0030599<br>GO:0005975<br>GO:0042545	aspartyl esterase activity<br>hydrolase activity, acting on glycosyl bonds<br>pectinesterase activity<br>carbohydrate metabolic process<br>cell wall modification

CMN_00250		pectinesterase	GO:0045330<br>GO:0016798<br>GO:0030599<br>GO:0005975<br>GO:0042545	aspartyl esterase activity<br>hydrolase activity, acting on glycosyl bonds<br>pectinesterase activity<br>carbohydrate metabolic process<br>cell wall modification
CMN_00321	*manX*	putative alpha-mannosidase	GO:0004559<br>GO:0030246<br>GO:0046872<br>GO:0006013	alpha-mannosidase activity<br>carbohydrate binding<br>metal ion binding<br>mannose metabolic process
CMN_00322	*bglY*	glycosyl hydrolase, family 4	GO:0004553<br>GO:0046872<br>GO:0016616<br>GO:0005975	hydrolase activity, hydrolizing O-glycosyl compounds<br>metal ion binding<br>oxidoreductase activity, acting on the CH-OH group of donors, NAD or NADP as acceptor<br>carbohydrate metabolic process
CMN_00385		esterase containing a GAF sensor domain		
CMN_Ps00018				
CMN_00425		putative oxidoreductase	GO:0016491	oxidoreductase activity
CMN_00469	*pheA*		GO:0004106<br>GO:0004664<br>GO:0009094	chorismate mutase activity<br>prephenate dehydratase activity<br>N-phenylalanine biosynthetic process
CMN_00470	*serS*		GO:0005737<br>GO:0005524<br>GO:0004828<br>GO:0016260<br>GO:0006434	cytoplasm<br>ATP binding<br>serine-tRNA ligase activity<br>selenocysteine biosynthetic process<br>seryl-tRNA aminoacylation
CMN_00476		conserved secreted protein		
CMN_00482		ABC transporter, ATPase component	GO:0005524	ATP binding

CMN_00486		transcriptional regulator, LacI family	GO:0003677<br>GO:0006355	DNA binding<br>regulation of DNA-templated transcription
CMN_00487		conserved hypothetical protein		
CMN_00541		NTP pyrophosphohydrolase	GO:0016787	hydrolase activity
CMN_00545	*hemE*	uroporphyrinogen decarboxylase	GO:0005737<br>GO:0004853<br>GO:0006782	cytoplasm<br>uroporphyrinogen decarboxylase activity<br>protoporphyrinogen IX biosynthetic process
CMN_00546	*hemY*		GO:0005737<br>GO:0004729<br>GO:0006783	cytoplasm<br>oxygen-dependent protoporphyrinogen oxidase activity<br>heme biosynthetic process
CMN_00667	*wzx2*	putative polysaccharide exporter	GO:0016020	membrane
CMN_00668	*wcnE*			
CMN_00708	*purC*	Phosphoribosylaminoimidazole-succinocarboxamidesynthase	GO:0005524<br>GO:0004639<br>GO:0006189	ATP binding<br>phosphoribosylaminoimidazolesuccinocarboxamide synthase activity<br>'de novo' IMP biosynthetic process
CMN_Ps00032				
CMN_00779	*wcoC*	undecaprenyl-phosphateglycosylphosphotransferase	GO:0016020<br>GO:0016740	Sugar transferases involved in lipopolysaccharide synthesis
CMN_00783	*wcoG*	putative cell surface protein	GO:0005975	carbohydrate metabolic process

CMN_00858	*ackA1*		GO:0005737<br>GO:0008776<br>GO:0005524<br>GO:0000287<br>GO:0006085<br>GO:0006082<br>GO:0016310	cytoplasm<br>acetate kinase activity<br>ATP binding<br>magnesium ion binding<br>acetyl-CoA biosynthetic process<br>organic acid metabolic process<br>phosphorylation
CMN_00865	*asdA*		GO:0004073<br>GO:0051287<br>GO:0050661<br>GO:0046983<br>GO:0071266<br>GO:0019877<br>GO:0009097<br>GO:0009089<br>GO:0009088	aspartate-semialdehyde dehydrogenase activity<br>NAD binding<br>NADP binding<br>protein dimerization activity<br>'de novo' L-methionine biosynthetic process<br>diaminopimelate biosynthetic process<br>isoleucine biosynthetic process<br>lysine biosynthetic process via diaminopimelate<br>threonine biosynthetic process
CMN_00928	*xthA*	exodeoxyribonuclease III	GO:0008311<br>GO:0006281	double-stranded DNA 3'-5' DNA exonuclease activity<br>DNA repair
CMN_01141		conserved hypothetical protein		
CMN_01143	*ogtB*	methylated-DNA-protein-cysteinemethyltransferase	GO:0005737<br>GO:0003908<br>GO:0006307<br>GO:0032259	cytoplasm<br>methylated-DNA-[protein]-cysteine S-methyltransferase activity<br>DNA dealkylation involved in DNA repair<br>methylation
CMN_01159	*pepP2*		GO:0030145<br>GO:0070006	manganese ion binding<br>metalloaminopeptidase activity
CMN_01186		hypothetical protein		
CMN_01187		conserved membrane protein	GO:0005886	plasma membrane

CMN_01357	*pyrH*		GO:0005737<br>GO:0005524<br>GO:0033862<br>GO:0044210<br>GO:0016310	cytoplasm<br>ATP binding<br>UMP kinase activity<br>'de novo' CTP biosynthetic process<br>phosphorylation
CMN_01359	*cdsA*	phosphatidate cytidylyltransferase	GO:0016020<br>GO:0004605<br>GO:0016024	membrane<br>phosphatidate cytidylyltransferase activity<br>CDP-diacylglycerol biosynthetic process
CMN_01443		monooxygenase	GO:0004497<br>GO:0016705	monooxygenase activity<br>oxidoreductase activity, acting on paired donors, with incorporation or reduction of molecular oxygen
CMN_01455		peptide ABC transporter, substrate-binding protein	GO:0043190<br>GO:0042597<br>GO:0055085	ATP-binding cassette (ABC) transporter complex<br>periplasmic space<br>transmembrane transport
CMN_01477		hypothetical protein	GO:0016020	membrane
CMN_01491		putative efflux MFS permease	GO:0016020<br>GO:0022857	membrane<br>transmembrane transporter activity
CMN_01500		nicotinamide mononucleotide transporter, PnuC family	GO:0016020<br>GO:0034257	membrane<br>nicotinamide riboside transmembrane transporter activity
CMN_01503		conserved hypothetical protein	GO:0008195	phosphatidate phosphatase activity
CMN_01591	*polA2*	DNA polymerase I	GO:0003677<br>GO:0003887<br>GO:0006974<br>GO:0006261	DNA binding<br>DNA-directed DNA polymerase activity<br>DNA damage response<br>DNA-templated DNA replication
CMN_01593		conserved hypothetical protein		

CMN_01599	*fabD*	malonyl CoA-acyl carrier protein transacylase	GO:0004314	[acyl-carrier-protein] S-malonyltransferase activity
CMN_01600		transcriptional regulator		
CMN_01603		conserved membrane protein	GO:0016020	membrane
CMN_01655	*xysB*	endo-1,4-beta-xylanase	GO:0110165<br>GO:0031176<br>GO:0045493	cellular anatomical entity<br>endo-1,4-beta-xylanase activity<br>xylan catabolic process
CMN_01889		FAD-binding molybdopterin dehydrogenase	GO:0071949<br>GO:0016491	FAD binding<br>oxidoreductase activity
CMN_01952	*tyrS*		GO:0005737<br>GO:0005524<br>GO:0003723<br>GO:0004831<br>GO:0006437	cytoplasm<br>ATP binding<br>RNA binding<br>tyrosine-tRNA ligase activity<br>tyrosyl-tRNA aminoacylation
CMN_01957	*argH*		GO:0005737<br>GO:0004056<br>GO:0042450	cytoplasm<br>argininosuccinate lyase activity<br>arginine biosynthetic process via ornithine
CMN_01961	*argB*		GO:0005737<br>GO:0003991<br>GO:0005524<br>GO:0042450<br>GO:0016310	cytoplasm<br>acetylglutamate kinase activity<br>ATP binding<br>arginine biosynthetic process via ornithine<br>phosphorylation
CMN_02086		conserved secreted protein		
CMN_02089		esterase		
CMN_02090		hypothetical membrane protein	GO:0016020	membrane

CMN_02127	*dxr*		GO:0030604<br>GO:0046872<br>GO:0070402<br>GO:0019288<br>GO:0016114	1-deoxy-D-xylulose-5-phosphate reductoisomerase activity<br>metal ion binding<br>NADPH binding<br>isopentenyl diphosphate biosynthetic process, methylerythritol 4-phosphate pathway<br>terpenoid biosynthetic process
CMN_02153		peptide ABC transporter, substrate-binding protein	GO:0043190<br>GO:0042597<br>GO:0055085	ATP-binding cassette (ABC) transporter complex<br>periplasmic space<br>transmembrane transport
CMN_02276		hypothetical secreted protein		
CMN_02302	*modA*	molybdate ABC transporter, substrate-binding protein	GO:0046872<br>GO:0015689	metal ion binding<br>molybdate ion transport
CMN_02325		DNase	GO:0004536	DNA nuclease activity
CMN_02353		membrane-bound dolichyl-phosphate-mannose-protein mannosyltransferase	GO:0005886<br>GO:0004169	plasma membrane<br>dolichyl-phosphate-mannose-protein mannosyltransferase activity
CMN_02389		methyltransferase	GO:0003676<br>GO:0008757<br>GO:0043412<br>GO:0032259<br>GO:0006807<br>GO:0044238	nucleic acid binding<br>S-adenosylmethionine-dependent methyltransferase activity<br>macromolecule modification<br>methylation<br>nitrogen compound metabolic process<br>primary metabolic process
CMN_02390	*cel*	secreted cellulase	GO:0008810<br>GO:0030247<br>GO:0030245	cellulase activity<br>polysaccharide binding<br>cellulose catabolic process
CMN_02404		conserved hypothetical protein		

CMN_02473	*galT*		GO:0008108<br>GO:0008270<br>GO:0033499	UDP-glucose:hexose-1-phosphate uridylyltransferase activity<br>zinc ion binding<br>galactose catabolic process via UDP-galactose
CMN_02474	*galK*		GO:0005737<br>GO:0005524<br>GO:0004335<br>GO:0000287<br>GO:0006012	cytoplasm<br>ATP binding<br>galactokinase activity<br>magnesium ion binding<br>galactose metabolic process
CMN_02503	*sucD*		GO:0000166<br>GO:0004775<br>GO:0004776<br>GO:0006099	nucleotide binding<br>succinate-CoA ligase (ADP-forming) activity<br>succinate-CoA ligase (GDP-forming) activity<br>tricarboxylic acid cycle
CMN_02505		conserved hypothetical protein	GO:0016491	oxidoreductase activity
CMN_02507	*uvrD4*	ATP-dependent DNA helicase	GO:0005524<br>GO:0016887<br>GO:0003677<br>GO:0003678	ATP binding<br>ATP hydrolysis activity<br>DNA binding<br>DNA helicase activity
CMN_02508	*glpQ1*	glycerophosphodiester phosphodiesterase	GO:0008889<br>GO:0006629	glycerophosphodiester phosphodiesterase activity<br>lipid metabolic process
CMN_02551		transcription antiterminator containing a PTS domain	GO:0006355	regulation of DNA-templated transcription
CMN_02587	*rpoB*	DNA-directed RNA polymerase subunit beta	GO:0000428<br>GO:0003677<br>GO:0003899<br>GO:0032549<br>GO:0006351	DNA-directed RNA polymerase complex<br>DNA binding<br>DNA-directed 5'-3' RNA polymerase activity<br>ribonucleoside binding<br>DNA-templated transcription
CMN_02589		conserved hypothetical protein		

CMN_02595		conserved hypothetical protein	GO:0050532<br>GO:0000287	2-phosphosulfolactate phosphatase activity<br>magnesium ion binding
CMN_02609		amidase	GO:0016020	membrane
CMN_02705		permease	GO:0005886	plasma membrane
CMN_02714	*kdpA*	potassium-transporting ATPase A chain, P-type ATPase	GO:0005886<br>GO:0016787<br>GO:0008556<br>GO:0030955	plasma membrane<br>hydrolase activity<br>P-type potassium transmembrane transporter activity<br>potassium ion binding
CMN_02717	*kdpD*	K+ channel two-component system sensor kinase	GO:0016020<br>GO:0005524<br>GO:0000155	membrane<br>ATP binding<br>phosphorelay sensor kinase activity
CMN_02719		putative secreted esterase/lipase		
CMN_02721		hypothetical membrane protein	GO:0016020	membrane
CMN_02723		putative alkylation damage DNA repair protein		
CMN_02795		homocysteine S-methyltransferase	GO:0008168<br>GO:0008270<br>GO:0009086<br>GO:0032259	methyltransferase activity<br>zinc ion binding<br>methionine bionsynthetic process<br>methylation
CMN_02815		putative zinc transporter, ZIP family	GO:0016020	membrane
CMN_02818		putative ATPase		
CMN_02822		conserved hypothetical protein		
CMN_02823		methyltransferase	GO:0008168<br>GO:0032259	methyltransferase activity<br>methylation

CMN_02853	*crtBI*	putative bifunctional phytoene synthase/phytoenedehydrogenase	GO:0004311 GO:0016491 GO:0016117	farnesyltranstransferase activity oxidoreductase activity carotenoid biosynthetic process
CMN_02884		conserved hypothetical protein		
CMN_02945	*rsmG*	ribosomal RNA small subunit methyltransferase G	GO:0005737 GO:0070043	cytoplasm rRNA (guanine-N7)-methyltransferase activity

Table S4. Loci under selection annotated by SnpEff to determine amino acid effects of SNPs within protein-coding regions. Of 202 SNPs within 94 loci, 77 code for missense variants within 51 individual loci. Locus tags are from strain NCPPB 2581 (GenBank Accession HE614873).

Locus	Variant Position	SnpEff Annotation
CMN_00039	337	missense variant
CMN_00047	375	synonymous variant
CMN_00050	255	synonymous variant
CMN_00051	81	synonymous variant
CMN_00051	468	missense variant
CMN_00051	504	synonymous variant
CMN_00051	537	synonymous variant
CMN_00065	765	synonymous variant
CMN_00065	766	missense variant
CMN_00065	767	missense variant
CMN_00065	768	synonymous variant
CMN_00065	1293	synonymous variant
CMN_00083	98	missense variant
CMN_00089	486	synonymous variant
CMN_00089	565	missense variant

CMN_00093	100	missense variant
CMN_00174	190	missense variant
CMN_00249	2179	missense variant
CMN_00249	2455	missense variant
CMN_00250	3931	missense variant
CMN_00321	2502	synonymous variant
CMN_00322	654	missense variant
CMN_00385	1258	missense variant
CMN_Ps00018	868	no annotation
CMN_00425	492	synonymous variant
CMN_00469	555	synonymous variant
CMN_00470	573	synonymous variant
CMN_00476	88	missense variant
CMN_00482	420	synonymous variant
CMN_00486	288	synonymous variant
CMN_00487	150	synonymous variant
CMN_00541	309	synonymous variant
CMN_00545	48	synonymous variant
CMN_00546	400	missense variant
CMN_00667	565	missense variant
CMN_00668	524	missense variant
CMN_00708	606	synonymous variant
CMN_Ps00032	1995	no annotation
CMN_00779	822	synonymous variant
CMN_00783	2354	no annotation
CMN_00783	3172	no annotation
CMN_00858	1025	missense variant
CMN_00865	387	synonymous variant
CMN_00928	24	synonymous variant
CMN_01141	315	synonymous variant

CMN_01143	418	missense variant
CMN_01159	1386	synonymous variant
CMN_01186	522	missense variant
CMN_01187	369	synonymous variant
CMN_01357	609	synonymous variant
CMN_01359	499	missense variant
CMN_01443	201	synonymous variant
CMN_01455	648	synonymous variant
CMN_01477	530	missense variant
CMN_01477	573	synonymous variant
CMN_01477	633	synonymous variant
CMN_01477	691	missense variant
CMN_01477	717	synonymous variant
CMN_01477	768	synonymous variant
CMN_01491	521	missense variant
CMN_01500	473	missense variant
CMN_01503	282	synonymous variant
CMN_01591	948	synonymous variant
CMN_01591	1116	synonymous variant
CMN_01593	132	missense variant
CMN_01599	243	synonymous variant
CMN_01600	198	synonymous variant
CMN_01603	420	synonymous variant
CMN_01655	1667	missense variant
CMN_01889	623	missense variant
CMN_01889	840	synonymous variant
CMN_01952	504	synonymous variant
CMN_01952	507	synonymous variant
CMN_01952	1136	missense variant
CMN_01957	177	synonymous variant

CMN_01957	300	synonymous variant
CMN_01957	322	missense variant
CMN_01957	342	synonymous variant
CMN_01957	354	synonymous variant
CMN_01957	441	synonymous variant
CMN_01957	443	missense variant
CMN_01957	528	synonymous variant
CMN_01957	531	synonymous variant
CMN_01957	573	synonymous variant
CMN_01957	639	synonymous variant
CMN_01961	156	synonymous variant
CMN_02086	428	missense variant
CMN_02089	738	synonymous variant
CMN_02090	194	missense variant
CMN_02090	195	synonymous variant
CMN_02090	213	synonymous variant
CMN_02090	216	synonymous variant
CMN_02090	228	synonymous variant
CMN_02090	232	missense variant
CMN_02090	233	missense variant
CMN_02127	871	missense variant
CMN_02153	1296	synonymous variant
CMN_02276	23	missense variant
CMN_02302	632	missense variant
CMN_02325	177	synonymous variant
CMN_02325	186	synonymous variant
CMN_02325	213	synonymous variant
CMN_02325	243	synonymous variant
CMN_02325	477	synonymous variant
CMN_02325	516	synonymous variant

CMN_02325	540	synonymous variant
CMN_02353	1566	synonymous variant
CMN_02389	1086	missense variant
CMN_02390	1306	missense variant
CMN_02404	1659	synonymous variant
CMN_02473	487	missense variant
CMN_02473	488	missense variant
CMN_02473	851	missense variant
CMN_02473	882	synonymous variant
CMN_02473	903	synonymous variant
CMN_02473	906	synonymous variant
CMN_02473	907	missense variant
CMN_02474	219	missense variant
CMN_02503	480	synonymous variant
CMN_02505	590	missense variant
CMN_02507	1431	synonymous variant
CMN_02508	433	missense variant
CMN_02551	1227	synonymous variant
CMN_02587	109	synonymous variant
CMN_02587	1813	synonymous variant
CMN_02589	639	synonymous variant
CMN_02595	247	missense variant
CMN_02609	718	missense variant
CMN_02609	1844	missense variant
CMN_02705	249	synonymous variant
CMN_02705	252	synonymous variant
CMN_02705	267	synonymous variant
CMN_02705	276	synonymous variant
CMN_02705	287	missense variant
CMN_02705	322	missense variant

CMN_02705	402	synonymous variant
CMN_02705	408	synonymous variant
CMN_02705	519	synonymous variant
CMN_02705	528	synonymous variant
CMN_02705	531	synonymous variant
CMN_02714	916	missense variant
CMN_02714	918	synonymous variant
CMN_02717	2028	synonymous variant
CMN_02717	2031	synonymous variant
CMN_02717	2043	synonymous variant
CMN_02717	2070	synonymous variant
CMN_02717	2136	synonymous variant
CMN_02717	2142	synonymous variant
CMN_02717	2247	missense variant
CMN_02717	2253	synonymous variant
CMN_02717	2265	synonymous variant
CMN_02719	258	synonymous variant
CMN_02719	263	missense variant
CMN_02719	303	synonymous variant
CMN_02719	321	synonymous variant
CMN_02719	327	synonymous variant
CMN_02719	357	synonymous variant
CMN_02719	358	missense variant
CMN_02719	360	synonymous variant
CMN_02719	363	synonymous variant
CMN_02719	369	synonymous variant
CMN_02719	381	synonymous variant
CMN_02719	543	synonymous variant
CMN_02719	544	missense variant
CMN_02719	545	missense variant

CMN_02719	570	synonymous variant
CMN_02719	582	synonymous variant
CMN_02719	604	missense variant
CMN_02719	641	missense variant
CMN_02719	651	synonymous variant
CMN_02719	666	synonymous variant
CMN_02719	702	synonymous variant
CMN_02719	726	synonymous variant
CMN_02719	735	synonymous variant
CMN_02719	768	synonymous variant
CMN_02721	12	missense variant
CMN_02721	16	missense variant
CMN_02721	18	synonymous variant
CMN_02721	26	missense variant
CMN_02721	72	synonymous variant
CMN_02721	90	synonymous variant
CMN_02721	93	synonymous variant
CMN_02721	94	missense variant
CMN_02721	102	synonymous variant
CMN_02723	894	synonymous variant
CMN_02723	895	missense variant
CMN_02723	896	missense variant
CMN_02723	936	synonymous variant
CMN_02723	939	synonymous variant
CMN_02723	972	synonymous variant
CMN_02723	1164	synonymous variant
CMN_02795	668	missense variant
CMN_02815	534	synonymous variant
CMN_02815	543	synonymous variant
CMN_02815	552	synonymous variant

CMN_02818	1720	missense variant
CMN_02822	339	synonymous variant
CMN_02823	487	missense variant
CMN_02853	1791	synonymous variant
CMN_02853	1798	missense variant
CMN_02884	205	missense variant
CMN_02945	33	synonymous variant

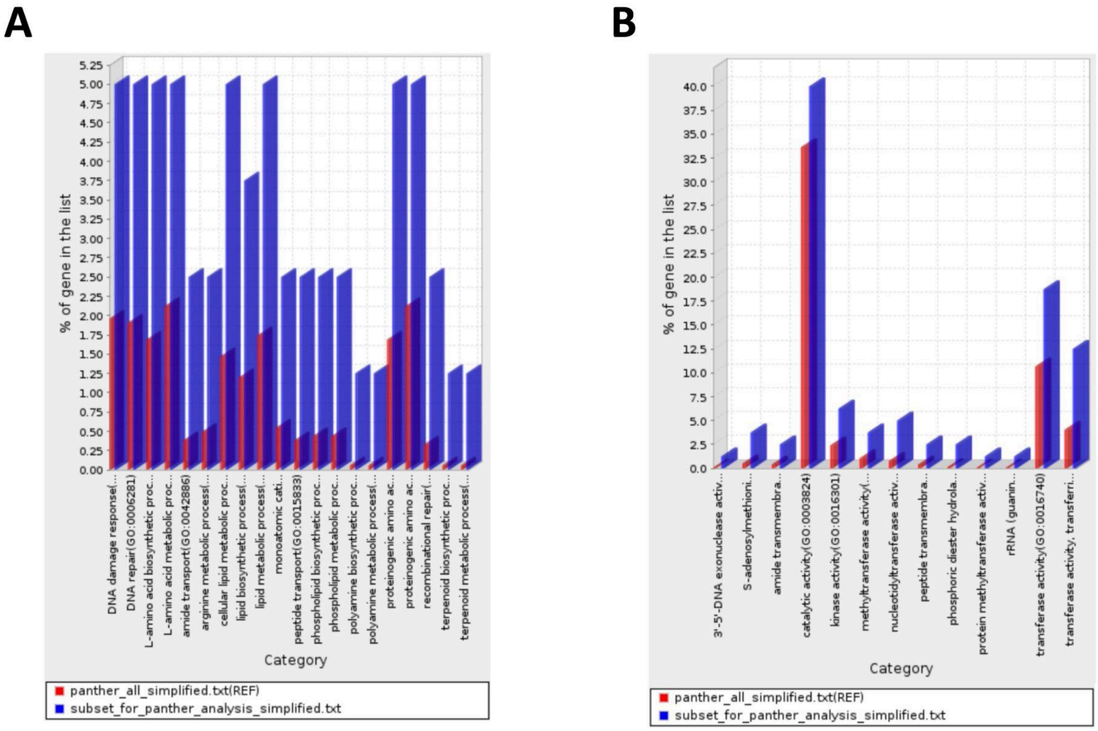

Figure S1. Output from PantherDB GO term enrichment analyses of genes under selection, showing biological process terms that were enriched (A) and molecular function terms that were enriched (B). No cellular component terms showed enrichment in the gene set.

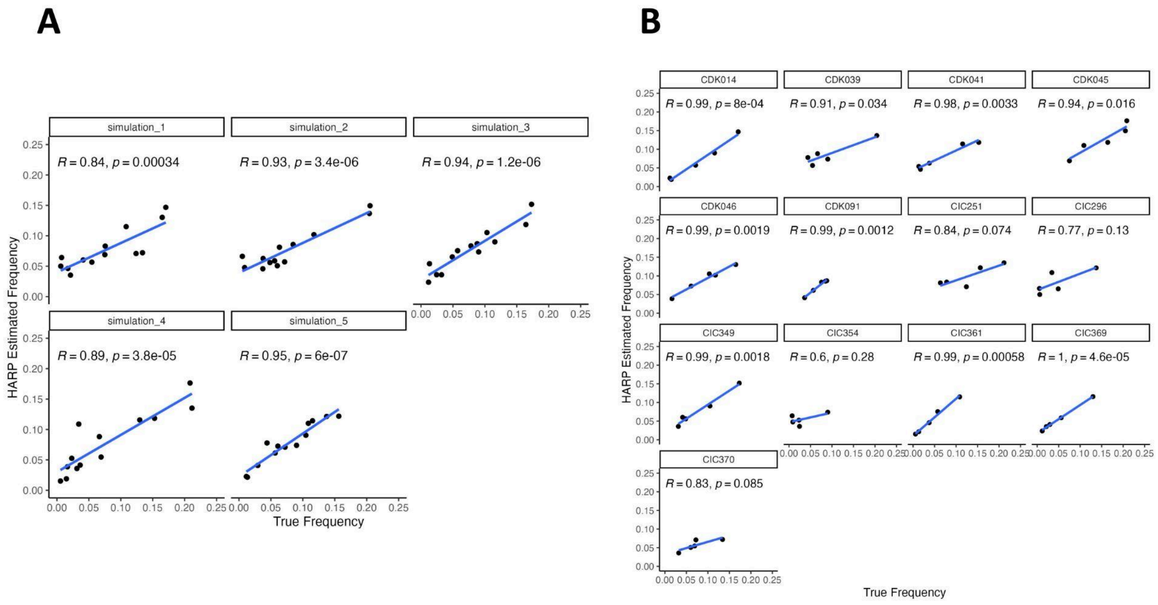

Figure S2. Correlation plots of true frequencies by estimated frequencies of sampled reads in five simulated pools by the software HARP. High correlations between true and estimated frequencies of strains are apparent within simulated pools (A) and on a strain basis between simulated pools (B).

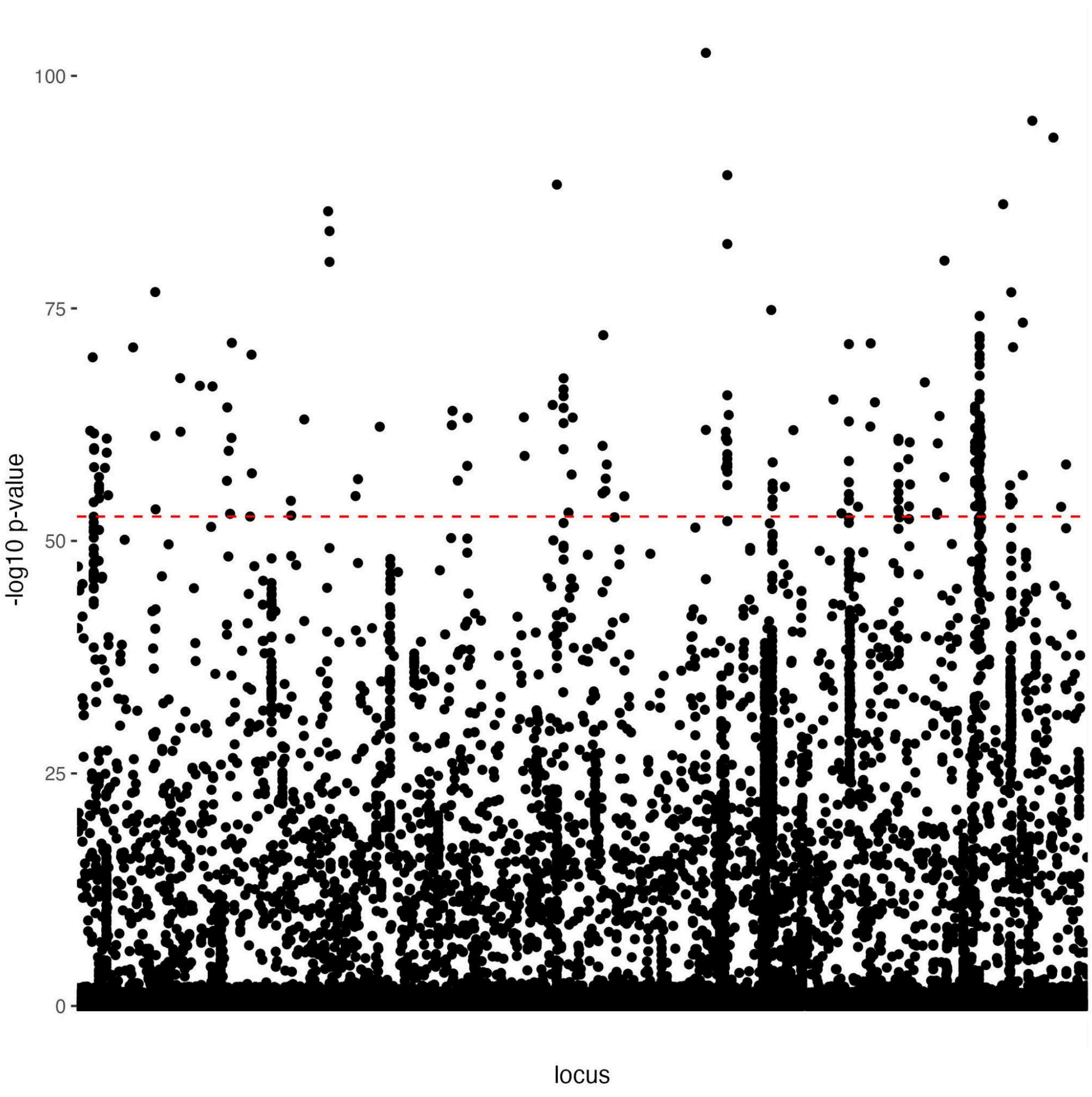

Figure S3. Manhattan plot of significant hits from Fisher's exact test of loci under selection in all pooled samples. Red line indicates the 99.5th percentile. A total of 202 SNPs in 94 loci were identified as hits above this threshold, and were considered top candidate genes in virulence of *Cn*.
